# The Cytoskeleton in Adrenal Physiology and Tumours: Functional Roles and Emerging Molecular Targets

**DOI:** 10.3390/ijms262110348

**Published:** 2025-10-24

**Authors:** Rosa Catalano, Emma Nozza, Emanuela Esposito, Sonia Di Bari, Giovanna Mantovani, Erika Peverelli

**Affiliations:** 1 Department of Clinical Sciences and Community Health, Department of Excellence 2023–2027, University of Milan, 20122 Milan, Italy; rosa.catalano@unimi.it (R.C.); emma.nozza@unimi.it (E.N.); emanuela.esposito1@unimi.it (E.E.); sonia.dibari@unimi.it (S.D.B.); 2 PhD Program in Experimental Medicine, University of Milan, 20122 Milan, Italy; 3 Endocrinology Unit, Fondazione IRCCS Ca’ Granda Ospedale Maggiore Policlinico, 20122 Milan, Italy

**Keywords:** cytoskeleton, cytoskeleton-binding proteins, adrenal cortex, steroidogenesis, adrenocortical carcinoma (ACC)

## Abstract

The cytoskeleton has been described as a regulator of adrenal physiology and tumour behaviour. In the adrenal cortex, both cytoskeletal filaments, by mediating cholesterol transfer to mitochondria, and their binding proteins, such as cofilin and diaphanous-related formin 1 (DIAPH1), have been implicated in modulating steroidogenic processes. Beyond hormone production, the cytoskeleton participates in oncogenic signalling and contributes to the acquisition of malignant behaviour in adrenocortical carcinoma (ACC). Cytoskeleton-associated proteins such as filamin A (FLNA), fascin-1 (FSCN1), RASSF1A, and the guanine nucleotide exchange factor VAV2 are involved in signal transduction, cell cycle regulation, and cytoskeletal remodelling. In ACC, dysregulation of the expression or activity of these proteins correlates with ACC aggressiveness, including increased proliferation, motility, and invasion as well as poor prognosis, making them attractive candidates for targeted therapeutic strategies. To date, no review has systematically addressed the role of cytoskeleton and its binding partners in both adrenal physiological regulation and pathological context. This review is the first to provide a comprehensive overview of cytoskeletal involvement in adrenal cortex function and cancer, highlighting emerging molecular players and their possible therapeutic implications.

## 1. Introduction

The cytoskeleton is a complex and dynamic intracellular structure that was historically considered as a mere static scaffold and later recognized as a dynamic structure capable of orchestrating a wide range of cellular processes and remodelling itself in response to intra and extracellular stimuli [1]. The cytoskeleton is composed of actin filaments (also known as microfilaments), intermediate filaments, and microtubules. Actin filaments and microtubules are made of globular proteins: actin and tubulin, respectively [2,3]. In contrast, intermediate filaments consist of a heterogeneous family of fibrous proteins including vimentin, desmin, cytokeratins, keratins, and lamins, whose expression is tissue and cell type specific. In the adrenal cortex, for instance, cytokeratins are predominantly expressed in normal tissue, but their levels decrease progressively in adrenocortical adenomas (ACA) and carcinomas (ACC), where vimentin becomes the dominant intermediate filament [4]. Each filament system contributes both structurally and functionally to cell biology. Actin filaments and microtubules participate in the maintenance of the cell shape and both provide structural support. They are involved in cell division and also serve as directional tracks for motor proteins enabling active intracellular trafficking [5]. In addition, actin filaments play a role in cell migration and endocytosis [2], while microtubules are essential for building cellular structures like the mitotic spindle and the axonemes of cilia and flagella [3]. Although actin filaments and microtubules have been classically viewed as separate players in processes like cell migration, mitosis, and intracellular trafficking, a recent study highlighted a coordinated crosstalk between them [6]. By contrast, intermediate filaments do not serve as tracks for motor proteins but are themselves transported along other cytoskeletal elements. In addition, they are involved in maintaining the cell shape, and giving mechanical reinforcement to the cytoplasm, as well as participating in the signal transduction [5].

The cytoskeleton functions are tightly regulated also by a wide range of binding proteins that actively control processes such as nucleation, polymerisation dynamics, crosslinking, severing, and anchoring of cytoskeletal structures [1]. Recent discoveries have highlighted how these binding proteins contribute to cell migration, signal transduction, vesicle trafficking, and even tumour progression, primarily through their modulation of cytoskeletal organization and dynamics. Among them, proteins such as filamin A, fascin, and cofilin, which interact with actin filaments, have gained particular attention for their roles in cytoskeletal plasticity and invasive behaviour in cancer cells [7].

The adrenal glands are two endocrine organs located on the top of each kidney, composed of an outer adrenal cortex and an inner medulla. The adrenal cortex derives embryologically from the mesoderm and is structurally divided into three zones that are named from the outermost to innermost, respectively, as follows: zona glomerulosa, zona fasciculata, and zona reticularis. Each zona synthesizes specific steroid hormones. The mineralocorticoids, produced by the zona glomerulosa, are responsible for blood pressure regulation and electrolyte balance. The glucocorticoids, synthesised by the zona fasciculata, are involved in glucose metabolism, anti-inflammatory response, and stress management. Androgens, secreted by the zona reticularis, play a role in the development of secondary sexual characteristics and in sexual function, as well as in muscle and bone growth [8]. Both glucocorticoid and androgen secretion is controlled by the adrenocorticotropic hormone (ACTH), produced by the pituitary, which acts to increase the intracellular level of the second messenger cyclic AMP (cAMP) that activates the cAMP/PKA pathway [9]. Alteration of cell proliferation within the adrenal cortex gives rise to a spectrum of pathological conditions including adrenocortical hyperplasia, adrenocortical nodular disease, ACA, and ACC [10]. ACA are common benign tumours, typically unilateral and often detected incidentally during imaging for unrelated conditions. While many are non-functioning, some ACA secrete hormones autonomously, causing clinical syndromes such as Cushing syndrome or primary aldosteronism [11]. Despite their benign nature, accurate hormonal evaluation and imaging are essential for appropriate classification, management, and exclusion of malignancy. ACC is a rare but aggressive malignancy, frequently presenting hormone excess, for which the surgical resection remains the treatment of choice. However, the risk of recurrence is high, and therapeutic options beyond surgery, such as mitotane and cytotoxic chemotherapy, remain limited and ineffective. As a result, the prognosis is poor, especially in advanced disease stages [12]. In recent years, numerous studies aimed to improve the accuracy of the ACC diagnosis, better identifying patients at high risk of recurrence, and to deepen the biological understanding of ACC [12]. A better understanding of the cellular and molecular biology of the adrenal cortex is essential to refine diagnostic approaches and develop targeted therapies. Among the key intracellular structures, the cytoskeleton has emerged as a critical player in regulating adrenal cortical cell architecture, trafficking, signal transduction, and tumour behaviour.

This review aims to summarise current knowledge on the roles of the cytoskeleton and its associated binding proteins in the physiological functions of adrenal cortical cells and their involvement in pathological processes, including tumourigenesis.

## 2. Role of the Cytoskeleton in Steroidogenesis and Adrenocortical Tumourigenesis

### 2.1. Cytoskeletal Regulation of Steroidogenesis in the Adrenal Cortex

**Figure 1:**
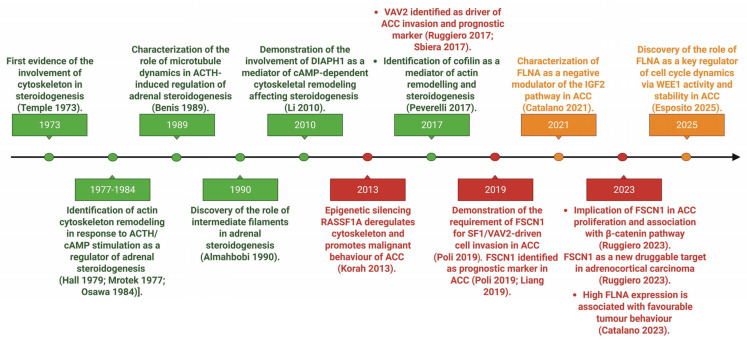
Timeline of key discoveries linking the cytoskeleton to adrenal physiology and pathology [13,14,15,16,17,18,19,20,21,22,23,24,25,26,27,28,29]. Green: Publications highlighting discoveries on the role of the cytoskeleton and its binding proteins in adrenal steroidogenesis. Orange: Studies investigating the involvement of the cytoskeleton-binding proteins in the regulation of oncogenic signalling in ACC. Red: Articles focusing on the impact of cytoskeletal binding protein on ACC behaviour.

In 1973, the pioneering work of Temple and Wolff proposed some of the first evidence for an involvement of the cytoskeleton in steroidogenesis [13] (Figure 1). The authors demonstrated that antimicrotubular agents such as colchicine, vinblastine, and podophyllotoxin markedly stimulated steroid production in mouse adrenocortical tumour Y-1 cells, in a manner comparable to ACTH stimulation but through a mechanism independent of the cAMP signalling pathway. They proposed that microtubule disruption facilitated mitochondrial access to cholesterol, hypothesizing for the first time that the cytoskeleton could regulate steroidogenesis by acting as a physical intracellular barrier.

**Figure 2:**
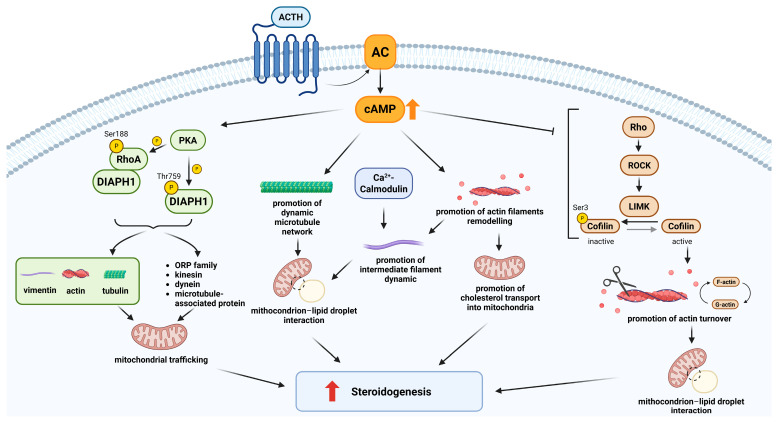
The role of the cytoskeleton in adrenal steroidogenesis. This figure schematically represents the involvement of cytoskeleton filaments and cytoskeleton binding proteins in steroidogenesis. The adrenocorticotropic hormone (ACTH) acts to increase the amount of cyclic AMP (cAMP) in adrenal cells. The activation of the cAMP pathway promotes both an increase in the microtubule dynamic and actin filament remodelling with a consequent increase in cholesterol transport into the mitochondria and a promotion of steroidogenesis. The ATP-dependent actomyosin contraction also has an effect on the reorganization of intermediate filaments, increasing mitochondria–lipid droplet interaction and steroidogenesis. Intermediate filament remodelling is also promoted by Ca^2+^-calmodulin phosphorylation. Two pathways involving cytoskeletal binding partners have been identified as key mediators of the effect of cAMP on the cytoskeleton in promoting steroidogenesis. cAMP activates PKA, which is able to phosphorylate RhoA on serine 188, promoting its interaction with diaphanous-related formin 1 (DIAPH1), and to directly phosphorylate DIAPH1 on threonine 759. In both cases, DIAPH1 is able to directly bind vimentin, actin, and tubulin as well as binding partners or cytoskeletal regulators, promoting cytoskeletal remodelling and consequently increasing mitochondrial trafficking and steroidogenesis. In addition, cAMP produces an inhibitory effect on the Rho-ROCK-LIMK pathway, reducing the phosphorylation and thus inactivation of cofilin. The presence of a more active cofilin leads to increased actin filament remodelling, promoting mitochondria–lipid droplet interaction and steroidogenesis.

At the end of the 1970s, two studies conducted in different experimental models provided complementary insights [30,31]. In primary rat adrenocortical cells, inhibition of both actin filaments and microtubules caused a marked decrease in steroidogenesis, suggesting a structural and essential role of the cytoskeleton in cholesterol trafficking to mitochondria [30]. In parallel, in Y-1 cells, the inhibition of actin polymerisation enhanced steroid production, again in a manner independent from ACTH [31]. A connection between the role of microfilaments in steroidogenesis and ACTH-mediated steroidogenic response via the cAMP pathway has been demonstrated by subsequent studies [14,15,16] (Figure 1). Specifically, the experiment conducted suggested a model in which ACTH, via the cAMP pathway, stimulates steroidogenesis by remodelling actin filaments facilitating cholesterol transport into mitochondrion (Figure 2 and Table 1).

The specific role of microtubules has been further explored in various models. In Y-1 cells, microtubule stabilisation with taxol significantly inhibited both basal and ACTH-stimulated steroidogenesis, suggesting that a dynamic microtubule network is required for efficient cholesterol transfer to mitochondria [17] (Figure 1 and Figure 2). Supporting this hypothesis, Nan and colleagues provided direct visual evidence. Using Coherent Anti-Stokes Raman Scattering (CARS) microscopy, they showed that lipid droplets (LDs) move actively along microtubules, and that this movement is enhanced by ACTH stimulation and suppressed by nocodazole-mediated microtubule disruption [32]. Increased LD mobility promotes contact with mitochondria, suggesting a functional role for active transport in cholesterol delivery. Consistent results were obtained in the human adrenocortical cell line H295R [18] (Figure 2). However, a study conducted in primary rat adrenocortical cells proposed a different mechanism [33]. While confirming that colchicine stimulates steroidogenesis, the authors demonstrated that this effect does not depend on microtubule disassembly but is instead linked to the removal of the protein coat surrounding LDs, thereby making esterified cholesterol accessible for mitochondrial conversion. Although apparently divergent, these two mechanisms may reflect complementary aspects of steroidogenesis: one involving cholesterol release from LDs, and the other its transport to mitochondria via active trafficking; both potentially modulated according to the cellular model and experimental context (Table 1).

The interest in the role of intermediate filaments in steroidogenesis emerged in the early 1990s. In Y-1 cells, it was shown that LDs are physically tethered to vimentin, with single LDs interacting with multiple filaments and vice versa, suggesting a three-dimensional structural anchoring network [19,34] (Figure 1). Subsequent studies in rat adrenocortical cells demonstrated a dynamic role for intermediate filaments. Upon Ca^2+^/calmodulin-mediated phosphorylation, vimentin filaments disassemble, facilitating the proximity of LDs and mitochondria [35] (Figure 2 and Table 1). This reorganization is accompanied by an ATP-dependent actomyosin contraction, mediated by phosphorylation of myosin light chain, resulting in cellular rounding (Figure 2 and Table 1). This morphological change is associated with increased steroid production, even in the absence of changes in cAMP levels [35]. The link between cell shape and steroidogenesis was clearly demonstrated [38]. In Y-1 cells, forskolin-induced rounding was shown to be associated with enhanced steroid production. This morphological remodelling is regulated by phosphorylation of paxillin, a focal adhesion protein, whose tyrosine dephosphorylation is required for steroidogenesis. Inhibition of tyrosine phosphatases blocked both cell rounding and hormone secretion, suggesting that cell shape remodelling represents a functionally relevant step closely linked to steroidogenic activity, even independently of cAMP signalling [38].

In vivo evidence supporting the involvement of intermediate filaments in steroidogenesis came from studies on vimentin knockout mice [39]. In this model, stimulation with ACTH or gonadotropins resulted in decreased production of corticosterone and progesterone, but not testosterone, suggesting a tissue-specific role for vimentin in the adrenal and ovary, but not in the testis. The absence of changes in steroidogenic enzyme expression or cholesterol uptake supports the conclusion that vimentin is essential for intracellular cholesterol trafficking to mitochondria.

Given the established role of cytoskeletal filaments in response to activation of the cAMP pathway, further investigations have aimed to elucidate the underlying intracellular signalling cascades and identify the cytoskeletal binding partners involved.

In H295R adrenocortical cells, stimulation with ACTH activates RhoA, a small GTPase of the Rho family involved in cytoskeletal remodelling, and its effector DIAPH1 (diaphanous-related formin 1). DIAPH1 is a member of the formin family of proteins that regulate cell and organelle motility and migration by interacting with both microtubules and actin filaments. The activation of the RhoA–DIAPH1 signalling axis promotes mitochondrial trafficking toward the perinuclear region, a process that facilitates the delivery of cholesterol to mitochondria and enhances cortisol synthesis [18] (Figure 1 and Table 1). Further studies demonstrated that it is the phosphorylation of DIAPH1 at threonine 759 (T759), by ERK or PKA, that regulates its stability and interaction with specific partners such as kinesin, actin, and oxysterol-binding protein–related protein 2 (ORP2), allowing proper mitochondrial movement [36]. Functional disruption of this pathway, either through DIAPH1 silencing, pharmacological inhibition of RhoA, or the transfection of a non-phosphorytable DIAPH1 mutant, impairs mitochondrial movement and leads to a marked reduction in cortisol production, underscoring the essential role of this axis in adrenal steroidogenesis [18,36] (Figure 2).

In parallel, cofilin has been identified as a critical mediator of actin remodelling and steroidogenesis in adrenocortical cells. Cofilin is an actin-binding protein that promotes actin filament depolymerisation and severing, both crucial processes for cytoskeletal reorganization [40]. Its activity is regulated by the phosphorylation of Ser3 by the Rho–Rho-associated kinase (ROCK)—LIM kinase (LIMK) signalling pathway. In Y-1 adrenocortical tumour cells and adrenocortical adenoma primary cells, stimulation with forskolin, a potent activator of adenylate cyclase and the cAMP pathway, induces cofilin dephosphorylation at Ser3, the active form of the protein. This modification is associated with actin rearrangement and consequent cell rounding and enhanced steroid hormone production (Figure 2). To demonstrate a causal role of cofilin activation in mediating forskolin effects, the authors used three approaches: (1) genetic silencing of cofilin, (2) transient transfection of wild-type or mutant cofilin (S3A, constitutively active, and S3D, phospho-mimetic inactive), and (3) pharmacological inhibition of cofilin phosphorylation. All approaches consistently showed that active cofilin is required for actin remodelling and steroidogenesis. In more detail, silencing of cofilin significantly impaired both actin remodelling and steroid secretion, and, in agreement, transfection of a constitutively active cofilin mutant (S3A) mimicked the effects of forskolin stimulation, confirming the direct involvement of cofilin in cAMP-mediated steroidogenesis [20]. Furthermore, using both Y-1 cells and primary adrenocortical cell cultures, the authors demonstrated that altering cofilin phosphorylation status, without an effect on total cofilin expression, using an inhibitor of ROCK, potentiated the cellular responses to the cAMP pathway activation [20]. Finally, the immunohistochemistry performed on human adrenal tissues showed that cortisol-producing adenomas (CPA) displayed a more active cofilin compared to the endocrine-inactive adenomas, supporting its role in steroidogenic function in vivo [20] (Figure 1 and Table 1).

These studies collectively demonstrate that both cytoskeletal filaments and their regulatory proteins directly influence key processes in steroidogenesis, highlighting the importance of a highly regulated and adaptable cytoskeletal network for proper steroidogenic function in adrenocortical cells.

### 2.2. Cytoskeleton Regulation of Oncogenic Signalling in Adrenocortical Carcinoma

In addition to supporting the steroidogenic function in adrenal cells, the cytoskeleton also works as a dynamic scaffold that modulates intracellular signalling pathways. This function has a significant impact on adrenocortical tumourigenesis, where alterations of the cytoskeleton can modify signal transduction, contributing to oncogenic progression.

Among the molecular pathways implicated in ACC pathogenesis, the insulin-like growth factor (IGF) system has been deeply studied. IGF2 is markedly overexpressed in about 80–90% of ACC compared to ACA and normal adrenal gland (NAG) [41,42,43,44]. By binding to the insulin-like growth factor 1 receptor (IGF1R) and to the insulin receptor isoform A (IRA), IGF2 signals through the mitogen-activated protein kinase (MAPK) and phosphatidylinositol-3-kinase (PI3K)/Akt pathways, boosting cell proliferation and survival [45]. Recently, the role of FLNA in modulating the IGF signalling pathway has been elucidated [21]. FLNA is a large actin-binding protein composed of 24 immunoglobulin-like repeats, ubiquitously expressed in humans, which crosslinks actin filaments. Beyond its structural role in actin cytoskeleton organization, FLNA acts as a multifunctional scaffold protein involved in cell adhesion, migration, and signal transduction. Through these roles, FLNA contributes to the spatial organization of receptor complexes and the modulation of intracellular signalling cascades [46,47,48].

**Figure 3:**
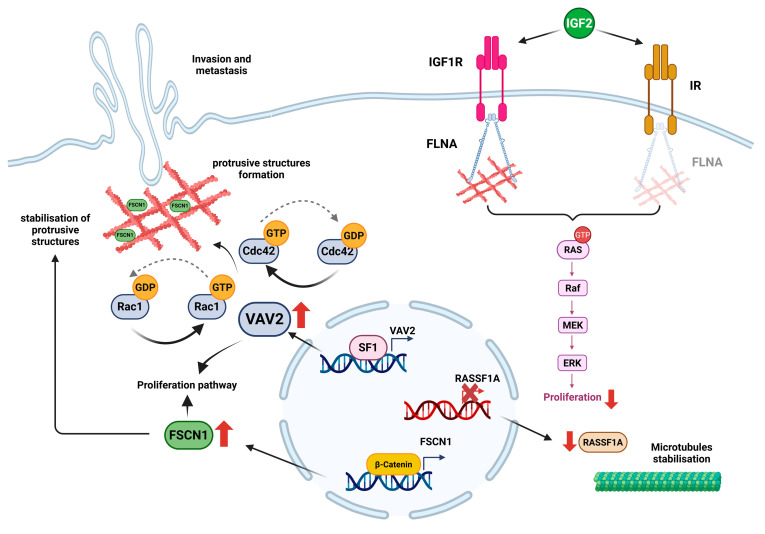
Cytoskeletal dynamics in adrenocortical carcinoma (ACC) cell invasion and proliferation. This figure schematically represents the involvement of cytoskeleton-actin binding proteins in ACC behaviour. The nuclear receptor steroidogenic factor 1 (SF-1), overexpressed in ACC, transcriptionally induces the nucleotide exchange factor VAV2. Once in the cytoplasm, VAV2 activates Rac1 and Cdc42, which drive lamellipodia and filopodia formation, respectively, promoting cell motility and invasion. In addition, VAV2 is associated with increased cell proliferation. The actin-binding protein fascin-1 (FSCN1) expression is regulated by β-catenin and is required for SF-1/VAV2-driven cell invasion. In more detail, FSCN1 stabilizes the protrusive structures, increasing invasiveness, and is associated with an increase in cell proliferation. The hypermethylation of the RASSF1A promoter reduces its expression and ability to stabilise microtubules and control mitotic spindle dynamics, resulting in a rise in cell proliferation, motility, and invasiveness. The expression of the actin-binding protein filamin A (FLNA) is downregulated in the majority of ACC. When present, FLNA binds both IGF1R and IR with a downregulation of the ERK pathway, reducing IGF2-mediated cell proliferation.

In human ACC cells, FLNA was shown to interact with both IGF1R and IR, and these interactions were modulated by IGF2 stimulation [21]. Specifically, IGF2-mediated activation of these receptors increased FLNA-IGF1R complexes, while decreasing FLNA-IR ones [21]. Of note, receptor expression itself appeared to be regulated by FLNA levels: its silencing led to augmented IGF1R expression and reduced IR expression. Based on these findings, the authors suggested that FLNA may play a role in receptor turnover, possibly by protecting IR from degradation or promoting IGF1R degradation [21]. Furthermore, FLNA was identified as a negative regulator of the IGF2-driven signalling cascade that promotes cell proliferation (Table 1). Knockdown of FLNA enhanced IGF2-mediated cell proliferation by phosphorylating ERK and increasing cyclin E1 expression (Figure 3). Additionally, the efficacy of linsitinib (dual IGF1R/IR inhibitor) and NVP-ADW742 (IGF1R-specific inhibitor) was influenced by FLNA. FLNA-silenced H295R cells displayed an enhanced sensitivity to both inhibitors, responding to lower drug concentrations [21]. In line with previous findings, Western blot analysis conducted on a small set of adrenocortical tumours revealed that the average FLNA expression in ACC was notably lower compared to ACA, suggesting that reduced FLNA levels contribute to increased IGF1R expression and diminished regulation of the ERK pathway, thereby facilitating IGF2-driven tumour growth [21]. This expression pattern was confirmed by a large European multicentric study, which reported a loss of FLNA in the majority of ACC cases [22]. Altogether, these findings reveal a crucial role of the cytoskeletal protein FLNA in downregulating the hyperactivated IGF2 mitogenic pathway in ACC (Figure 1).

Beyond its role in IGF2 signalling, FLNA also regulates cell cycle progression in ACC through its interaction with the nuclear kinase Wee1, a key G2/M checkpoint regulator (Figure 1). In mouse neural progenitor cells, FLNA was shown to interact with Wee1 protein, altering its expression and activity [49]. At G2/M, Wee1 selectively inactivates cyclin-dependent kinase 1 (CDK1)-cyclin B1 complex activity by phosphorylating CDK1 at the Tyr15 residue, preventing premature mitotic entry with unrepaired or incompletely replicated DNA [50]. As reported by the authors, loss of FLNA function leads to increased Wee1 expression, delaying the onset and progression through mitosis [49]. In ACC, this regulatory role was further investigated. Protein expression analyses in tumour and normal adrenal samples revealed elevated Wee1 and reduced FLNA levels in ACC compared to normal adrenal tissues [23]. In the MUC-1 ACC cell line, FLNA knockdown increased Wee1 expression and activity, as shown by higher levels of phospho-CDK1 and cyclin B1, while these effects were reversed when FLNA was reintroduced. These results suggest that FLNA may promote Wee1 proteasomal degradation, as Wee1 downregulation was rescued by the proteasomal inhibitor lactacystin. Moreover, increased levels of phosho-Wee1 (Ser123), the first residue to be phosphorylated when Wee1 is sent to degradation, was reported in MUC-1 cells after FLNA transfection [23] (Table 1). Functionally, the Wee1 inhibitor AZD1775 reduced proliferation and induced DNA damage in ACC cells, with FLNA-silenced cells displaying an increased drug sensitivity [23]. These findings support Wee1 as a promising therapeutic target in ACC, particularly in those tumours lacking FLNA, where upregulated Wee1 levels may contribute to unchecked cell cycle progression. Collectively, this evidence reinforces the role of FLNA not only as a modulator of proliferative signalling, but also as a key player in cell cycle dynamics through regulation of Wee1 activity and stability.

### 2.3. Implications of Cytoskeletal Remodelling in the Aggressiveness of Adrenocortical Carcinomas

While modulating oncogenic signalling pathways, cytoskeletal alterations simultaneously contribute to tumour progression. Cellular architecture and polarity are often compromised by cytoskeletal disruption, enabling malignant features such as increased motility, invasiveness, and metastatic spread.

In adrenocortical carcinoma, the steroidogenic factor 1 (SF-1), a nuclear receptor pivotal for adrenal development and steroidogenesis, also plays a role in tumour invasiveness and proliferation. SF-1 overexpression is frequently observed in ACC [51,52,53] and it has been shown to transcriptionally induce VAV2, a guanine nucleotide exchange factor (GEF) for small Rho GTPases, in a dose-dependent manner [24] (Figure 1). Overexpressed VAV2 activates Rac1 and Cdc42, two key cytoskeletal regulators that drive lamellipodia and filopodia formation, respectively. These actin-rich protrusions are reinforced by focal adhesion complexes containing vinculin, paxillin, and focal adhesion kinase (FAK), promoting directional motility and invasion [24,37,54]. The presence of the SF-1–VAV2 axis illustrated here was demonstrated in H295R cells, where SF-1-induced invasion was abrogated by VAV2 silencing [24]. In addition, VAV2 expression in ACC positively correlates with the proliferation marker Ki67 and it was associated with poor prognosis [25] (Figure 1 and Figure 3) (Table 1).

A further effector regulated by SF-1 and functionally implicated in cytoskeletal remodelling and cell migration in ACC is fascin-1 (FSCN1). FSCN1 is an actin-bundling protein that stabilizes protrusive structures such as filopodia, lamellipodia, and invadopodia, allowing cell adhesion, motility, migration, and invasion [55] (Figure 3). FSCN1 protein presence was investigated in different tumour types, and it was correlated with increased risk of disease progression, metastasis, and mortality [55,56,57]. In adrenocortical carcinoma, FSCN1 is required for SF-1/VAV2-driven cell invasion [26] (Figure 1). The silencing or inhibition of FSCN1 suppressed the invasive phenotype induced by SF-1 and VAV2 overexpression. While VAV2 overexpression could partially rescue the loss of FSCN1, the opposite was not true, suggesting that VAV2 acts upstream by promoting Cdc42 and Rac1 activation and actin polymerisation, whereas FSCN1 is required to stabilize the resulting actin bundles (Figure 3 and Table 1). This sequential activation supports a model in which cytoskeletal remodelling and increased invasiveness depend on both effectors in a coordinated manner [26]. FSCN1 was also shown to be transcriptionally regulated by β-catenin, a key effector of the Wnt/Wingless signalling pathway encoded by the CTNNB1 gene, which is a frequently altered oncogenic driver in ACC [27] (Figure 1 and Figure 3). FSCN1 was found to be upregulated in the H295R cell line, which harbours an activating mutation in CTNNB1, as well as in patient samples with mutations in CTNNB1 or in other Wnt/β-catenin pathway components. Moreover, FSCN1 expression is positively correlated with both CTNNB1 levels and β-catenin target genes expression, suggesting a regulatory interplay between Wnt signalling and cytoskeletal dynamics [27] (Table 1). Functionally, Ruggiero et al. showed that FSCN1 is not only essential for the pro-migratory phenotype but also supports tumour proliferation (Figure 3). In H295R cells, inhibition or silencing of FSCN1 disrupted filopodia formation and reduced proliferation, and in zebrafish xenograft models, FSCN1 knockdown impaired both tumour growth and metastatic spread [27] (Figure 1). This dual regulation by SF-1 and β-catenin highlights the central role of FSCN1 in promoting the malignant phenotype of ACC. This molecular relevance is further supported by clinical evidence linking FSCN1 expression to key invasiveness parameters, including sinusoid, venous, and capsular invasion [26], and to poor prognosis, as indicated by reduced disease-free survival (DFS), overall survival (OS), and increased metastatic dissemination [26,28] (Figure 1 and Table 1).

However, cytoskeletal components can also exert tumour-suppressive functions, as exemplified by FLNA. This protein has been described as having a dual role in tumours, acting either as an oncogenic or tumour-suppressive factor depending on its subcellular localization, post-translational modification (as phosphorylation at Ser2125 or cleavage), and interaction with binding partners [46,58]. As previously described, our group has demonstrated the suppressive role of FLNA in ACC, showing that its low expression enhances IGF2-mediated proliferation [21] (Figure 3). A subsequent multicentre study, conducted on a large cohort of ACC patients (n = 119), identified a subset with a percentage of FLNA-positive cells higher than 5%, labelled as a “high-FLNA group”, which was associated with less aggressive features. Indeed, the “high-FLNA” group presented with a lower ENSAT stage, Weiss score, and mitotic index, as well as a higher rate of complete surgical resection, more favourable S-GRAS scores, and improved overall survival compared to the low FLNA group (Figure 1 and Table 1). These data support the hypothesis that FLNA loss potentiates IGF2 mitogenic effects, contributing to a more aggressive tumour phenotype and poorer outcome [22] (Figure 3).

A similar tumour-suppressive mechanism has been described for RASSF1A, a cytoskeleton-associated scaffold protein frequently silenced by promoter hypermethylation in ACC [29]. Reduced RASSF1A expression has been reported in ACC tissues and cell lines compared to adrenocortical adenomas and NAG [29]. Physiologically, RASSF1A stabilizes microtubules and controls mitotic spindle dynamics, thereby limiting cell proliferation, motility, and invasiveness. Re-expression of wild-type RASSF1A in ACC cells was able to revert malignant features, highlighting the critical role of cytoskeletal integrity in restraining tumour progression [29] (Figure 1 and Figure 3) (Table 1).

Altogether, these findings propose cytoskeletal remodelling as a fundamental driver of ACC aggressiveness, influencing both invasive capacity and proliferative behaviour. The molecular effectors described, VAV2, FSCN1, and FLNA, have been recognized for their clinical significance in ACC and, for this reason, proposed as potential biomarkers. When integrated with established clinical and molecular indicators, they might substantially improve ACC diagnostic precision and prognostic accuracy. VAV2 has been proposed, in combination with Ki67, to enhance patient stratification and give a more reliable prognostic assessment [25,59]. FSCN1, which correlates with multiple histopathological parameters of invasion and metastatic spread, has also been detected in circulation, making this protein a promising candidate for non-invasive pre-surgical evaluation [60]. Conversely, FLNA, considered as a potential tumour suppressor, could enhance diagnostic accuracy and prognostic stratification in ACC when evaluated alongside other clinical and molecular markers, offering significant prognostic value [22].

### 2.4. Therapeutic Approaches Targeting Cytoskeletal Functions: From Steroidogenesis to Tumour Aggressiveness

In the previous sections of this review, we highlighted the multifaceted role of the cytoskeleton in adrenal physiology and tumourigenesis, including its involvement in steroidogenesis, signalling, and tumour aggressiveness. These insights have prompted interest in the cytoskeleton as a possible target for pharmacological intervention; however, no cytoskeleton-based strategies have yet progressed beyond preclinical investigation.

In the context of steroidogenesis, although a number of pharmacological inhibitors, such as vinblastine, colchicine, and cytochalasin B, have been shown to disrupt cytoskeletal integrity and impair hormone secretion in vitro, as previously discussed in Section 2.1, these approaches remain limited to experimental settings. However, these studies may pave the way for future research to assess the cytoskeleton as a therapeutic target in the context of hormonally active adrenal adenomas, where dysregulated steroid hormone production represents a therapeutic challenge.

In oncogenic contexts, new therapeutic targets for ACC management are received with keen interest since effective options are currently limited to mitotane and only for advanced stage patients. Previously, we described FLNA as a modulator of Wee1 in ACC. Wee1 has emerged as a promising druggable target in several cancers, particularly in TP53-deficient cancers such as ovarian, triple-negative breast, and non-small cell lung cancer where its inhibition has shown promising results [61]. Our group recently demonstrated that the inhibition of Wee1 kinase, via AZD1775, significantly reduced cell viability in ACC models and that this effect was enhanced in the absence of FLNA, suggesting a potential interaction between cytoskeletal regulation and DNA damage checkpoint control [23]. However, despite these promising in vitro results, in vivo validation is still lacking, and further studies are needed to assess the therapeutic value of Wee1 inhibition in ACC. In addition, elucidating the functional interplay between FLNA and Wee1 in vivo could provide important insights into cytoskeleton-mediated control of cell cycle regulation and contribute to supporting the development of targeted therapy for ACC.

As previously discussed in Section 2.3, cytoskeletal remodelling also contributes to malignant behaviour through the SF-1/VAV2 axis. This pathway, involving downstream effectors Rac1 and Cdc42, promotes filopodia and lamellipodia formation, deputed to enhancing cell migration and invasiveness. From a therapeutic point of view, this interaction may represent a suitable target. VAV2/Rac1 interaction can be disrupted by EHop-016, a small molecule shown to inhibit Rac1 activity at 1 µM and Cdc42 at higher concentrations (>10 µM) [62,63]. Furthermore, its derivative MBQ-167 demonstrates greater potency and has been shown to markedly impair migration and prevent metastases formation in breast cancer models [64]. However, to date, none of these compounds have been tested in ACC preclinical models or patients.

Among cytoskeletal effectors involved in tumour aggressiveness, FSCN1 has emerged as an attractive therapeutic target due to its differential expression in normal versus tumoural adrenocortical tissues. This duality makes FSCN1 particularly suitable for selective inhibition. Various classes of FSCN1 inhibitors have been identified and tested in different cancer models: migrastatin derivatives such as macroketone, which bind at the actin-interacting domain of FSCN1 [57]; thiazole derivatives designed for FSCN1 inhibition that also exhibit antiangiogenic properties [65]; and miRNA-145, shown to downregulate FSCN1 expression through direct interaction with its 3′-UTR [66]. However, none of these agents has been successfully translated into clinical use for ACC. Of particular interest, the small molecule compound G2 and its optimized analogues have demonstrated the ability to reduce cancer cell migration and invasion by disrupting filopodia formation both in vitro and in vivo. Among them, NP-G2-044 is currently under clinical evaluation for advanced and metastatic solid tumours [56]. In the specific context of ACC, NP-G2-044 has been tested in vitro on FSCN1-expressing ACC cell lines and in a zebrafish model of metastatic ACC, resulting in decreased invasion and metastatic spread, respectively [27] (Figure 1). These findings position NP-G2-044 as the most promising FSCN1-targeting strategy currently available for potential applications in ACC patients.

Among cytoskeletal interactors, another tumour suppressor with therapeutic relevance is RASSF1A. As we explained in Section 2.3, the epigenetic silencing observed in ACC correlated with an increased cell proliferation, migration, and cytoskeletal disorganization. Although no direct pharmacological activators of RASSF1A are currently available, epigenetic therapies aimed at restoring its expression, such as DNA methyltransferase (DNMT) inhibitors, have shown efficacy in reactivating RASSF1A in other tumour types [67,68]. These agents could represent a potential therapeutic strategy in ACC, especially in the subset of patients with confirmed RASSF1A silencing.

## 3. Conclusions and Future Directions

This is the first review that provides a comprehensive overview of the role of the cytoskeleton and its binding partners in both adrenal physiological and pathological conditions. Cytoskeleton filaments and their binding proteins have emerged as important regulators of adrenal physiology and tumourigenic pathways. In the adrenal cortex, the remodelling of the cytoskeletal filaments is essential for coordinating cholesterol mobilization and transfer to mitochondria, promoting steroidogenesis. The demonstrated involvement of the binding proteins DIAPH1 and cofilin, in mediating these effects on hormone production, further highlights the importance of the cytoskeleton’s role in steroidogenesis. However, additional studies are needed to dissect the pathways involved, with the aim to have a deeper understanding of the molecular mechanism at the root of steroidogenic dysregulation and to identify possible molecular targets that could be targeted in the future by drugs, alone or in combination with the currently used steroidogenic inhibitors. Interestingly, the literature over the last few years has shown an involvement of cytoskeletal binding proteins in the tumour biology of ACC, with an influence in proliferative signalling, cell cycle regulation, and invasive behaviour. Some of these, such as VAV2 and FSCN1, have already been investigated as targeted therapies in adrenocortical carcinoma. However, the promising results are currently in the preclinical stage. Continued efforts to unravel these mechanisms may contribute to the development of more effective and targeted approaches for ACC.

## Figures and Tables

**Table 1:** Cytoskeletal and associated proteins in the adrenal cortex: summary of roles and clinical significance.

Protein	Level of Expression	Role	Clinical Significance	References
Actin filaments	/	ACTH–cAMP-mediated actin remodelling promotes cholesterol transport and steroidogenesis.	Modulation of steroidogenesis	[14,15,16]
Microtubules	/	ACTH-mediated dynamic of microtubule network is required for efficient cholesterol transfer to mitochondria.	Modulation of steroidogenesis	[17,18,32,33]
Intermediate filaments	/	Ca^2+^/calmodulin-dependent phosphorylation and actomyosin-driven contraction induces vimentin filament disassembly, promoting lipid droplet–mitochondria interaction.	Modulation of steroidogenesis	[19,34,35]
DIAPH1	/	Activated by the ACTH-cAMP-PKA axis, it promotes mitochondrial trafficking, facilitating cholesterol delivery and cortisol synthesis.	Modulation of steroidogenesis	[18,36]
Cofilin	Upregulated in cortisol-producing adenomas	Activated via Rho–ROCK–LIMK signalling, cofilin regulates actin turnover and promotes mitochondrion–lipid droplet interaction, supporting steroidogenic activity.	Modulation of steroidogenesis	[20]
FLNA	Downregulated in ACC	Negative regulator of the IGF2-induced proliferation pathway. Cell cycle regulator, FLNA promotes proteasomal degradation of Wee1 and premature mitotic entry. High FLNA expression correlates with less aggressive disease and better prognosis.	Anti-tumourigenic	[21,22,23]
VAV2	Upregulated in ACC	SF-1-dependent VAV2 upregulation promotes ACC invasion via Rac1 and Cdc42 activation. VAV2 positively correlates with Ki67 and negatively correlates with patients’ prognosis.	Pro-tumourigenic and metastatic	[24,25,37]
FSCN1	Upregulated in ACC	Determines a pro-migratory and invasive behaviour acting downstream of SF-1/VAV2 axis, stabilizing cell protrusions. FSCN1 expression is regulated by β-catenin and correlates with the expression of β-catenin target genes. High FSCN1 expression correlates with invasive features and poor prognosis.	Pro-tumourigenic and metastatic	[26,27,28]
RASSF1A	Downregulated in ACC	Microtubules stabilizer with anti-proliferative and anti-motility role.	Anti-tumourigenic	[29]

Acronym: DIAPH1, diaphanous-related formin 1; FLNA, filamin A; VAV2, Vav guanine nucleotide exchange factor 2; SF-1, Steroidogenic factor 1; FSCN1, fascin-1; and RASSF1A, Ras association domain family 1, isoform A.

## Data Availability

No new data were created or analysed in this study.

## Abbreviations

The following abbreviations are used in this manuscript:

DIAPH1	Diaphanous-related formin 1
ACC	Adrenocortical carcinoma
FLNA	Filamin A
FSCN1	Fascin-1
RASSF1A	Ras association domain family 1, isoform A
VAV2	Vav guanine nucleotide exchange factor 2
ACA	Adrenocortical adenoma
ACTH	Adrenocorticotropic hormone
cAMP	Cyclic AMP
PKA	Protein kinase A
CARS	Coherent Anti-Stokes Raman Scattering
LD	Lipid droplet
ATP	Adenosine triphosphate
RhoA	Ras homolog family member A
ROCK	Rho-associated kinase
LIMK	LIM kinase
CPA	Cortisol-producing adenoma
IGF	Insulin-like growth factor
NAG	Normal adrenal gland
IGF1R	Insulin-like growth factor 1 receptor
IRA	Insulin receptor isoform A
MAPK	Mitogen-activated protein kinase
PI3K	Phosphatidylinositol-3-kinase
SF-1	Steroidogenic factor 1
FAK	Focal adhesion kinase
DFS	Disease-free survival
OS	Overall survival
DNMT	DNA methyltransferase
